# Entrustment in physician-patient communication: a modified Delphi study using the EPA approach

**DOI:** 10.1186/s12909-021-02931-1

**Published:** 2021-09-20

**Authors:** Ayesha Younas, Rehan Ahmed Khan, Raheela Yasmin

**Affiliations:** 1 Department of Medical and Dental Education, Shifa College of Dentistry, Shifa Tameer-e-Millat University, Islamabad, Pakistan; 2 Islamic International Medical College, Riphah International University, Rawalpindi, Pakistan; 3 RARE Department, Islamic International Medical College, Riphah International University, Rawalpindi, Pakistan

**Keywords:** Assessment of communication, Competency based education, communication curriculum, Entrustable professional activities, Postgraduate studies, Modified Delphi study, Physician-patient communication

## Abstract

**Background:**

Competency based curricula across the globe stress on the importance of effective physician patient communication. A variety of courses have been used to train physicians for this purpose. However, few of them link competencies with practice resulting in confusions in implementation and assessment. This issue can be resolved by treating certain specific patient communication related tasks as acts of entrustment or entrustable professional activities (EPAs). In this study, we aimed to define a competency-based framework for assessing patient physician communication using the language of EPAs.

**Methods:**

A modified Delphi study was conducted in three stages. The first stage was an extensive literature review to identify and elaborate communication related tasks which could be treated as EPAs. The second stage was content validation by medical education experts for clarity and representativeness. The third stage was three iterative rounds of modified Delphi with predefined consensus levels. The McNemar test was used to check response stability in the Delphi Rounds.

**Results:**

Expert consensus resulted in development of 4 specific EPAs focused on physician-patient communication with their competencies and respective assessment strategies all aiming for level 5 of unsupervised practice. These include Providing information to the patient or their family about diagnosis or prognosis; Breaking Bad news to the patient or their family; Counseling a patient regarding their disease or illness; Resolving conflicts with patients or their families.

**Conclusions:**

The EPAs for Physician-patient communication are a step toward an integrative, all-inclusive competency-based assessment framework for patient-centered care. They are meant to improve the quality of physician patient interaction by standardizing communication as a decision of entrustment. The EPAs can be linked to competency frameworks around the world and provide a useful assessment framework for effective training in patient communication. They can be integrated into any post graduate curriculum and can also serve as a self-assessment tool for postgraduate training programs across the globe to improve their patient communication curricula.

**Supplementary Information:**

The online version contains supplementary material available at 10.1186/s12909-021-02931-1.

## Background

Almost a quarter into the twenty-first century, advances in technology have changed not only the way medicine is taught [1–3] but also, the way it is delivered to patients [4]. It would not be incorrect to say that healthcare education and delivery have been revolutionized in the past few decades [5, 6]. However, one core aspect of healthcare delivery remains traditional and occurs millions of times every day in every physician patient encounter: One conversation at a time. These conversations are actually the most frequently executed medical procedures [7–9] and their results may contribute to a large fraction of healthcare utilization [10]. Training students and residents how to partake in these conversations and effectively communicate with patients is now a necessity required by various accrediting bodies [11, 12]. Expertise in interpersonal and communication skills is expected at all levels of medical education. A review of the literature provides evidence of numerous communication curricula [13–15], which use various pedagogies and assessment modalities to develop and foster physician-patient communication. Nonetheless, all these programs are individual or institutional attempts to assess students and trainees for a skill that is universal, and as yet the medical profession has yet to agree on standard procedures or validated tools that may be used for the teaching and assessment of communication skills of the physician with the patient in any undergraduate or post graduate medical training program [16, 17].

Around the end of the last century, medical education witnessed a swift shift from the outcomes- based approach for medical curricula toward the development of competencies, giving ascent to the Competency Based Medical Education (CBME) movement [18]. The basic philosophy underlying CBME was the formulation of a set of competencies or predefined abilities as the outcomes of curricula [19]. CBME provided a shift in prominence away from time-based curricula in favor of needs-based graduate outcomes which were learner centered. Various competency-based frameworks for under and postgraduate medical students were introduced worldwide and over the span of the last two decades, literature both propagating and criticizing CBME has been published [20]. Of the various criticisms of CBME, one of the most widely discussed was the inability of programs worldwide to transform competencies into daily tasks resulting in confusion around their implementation and assessment. Varied implementations of CBME based programs around the globe failed to link the training of medical professionals to their practice [21]. A student can acquire a set of competencies but may be incapable of incorporating them into explicit tasks essential for adept performance.

To counter this claim, the concept of ‘entrustable’ professional activities (EPAs) was proposed by Olle Ten Cate in 2005 [22] with the aim of operationalizing CBME in post graduate programs. The EPA concept proposes combining various domains of competence to create an act of entrustment, thus bridging the gap between theoretical ideas of competence and realities of assessment in clinical practice [21]. Although initially proposed only for postgraduate training, the EPA concept is now more commonly applied in health professions education. Currently, EPAs are an integral part of many international medical curricula and have been developed for various subjects and themes covering various postgraduate specialties and undergraduate curricula [23]. EPAs unify various competencies to form descriptors of work that must be performed by the physician [24].

Professionalism and communication are necessary skills that are expected of all physicians and are included as competencies related to attitudes in almost all EPAs [25]. Yet, there are some specific tasks performed during routine physician – patient interaction that require only effective communication with the patient. Physician -patient communication literature shows that these skills should ideally be assessed by multiple assessors at multiple times throughout the continuum of clinical training [26]. This method of assessment aligns with the EPA concept of entrustment by using multiple assessors multiple times along with personalized learning [23]. The case for utilizing the concept of entrustment for verbal procedures was also proposed by Henry & colleagues [10]. Using an EPA framework for various physician patient communication related tasks will thus enable clinicians to address these communication tasks as recognizable professional activities performed in daily routine work which can easily be assessed by faculty [27]. No attempt has been made as yet to define these specific physician patient communication tasks as acts of entrustment. Our aim in this study was to develop EPAs for physician patient communication by expert consensus, along with their competencies, assessment strategies and supervision levels focusing on the entrustment of patient communication. Two research questions were developed according to our aims: 1. What are the desired EPAs for effective physician-patient communication? 2. What are the respective competencies (knowledge, skills and attitudes), assessment strategies and supervision levels needed for designing physician-patient communication EPAs?

## Methods

**Fig. 1:**
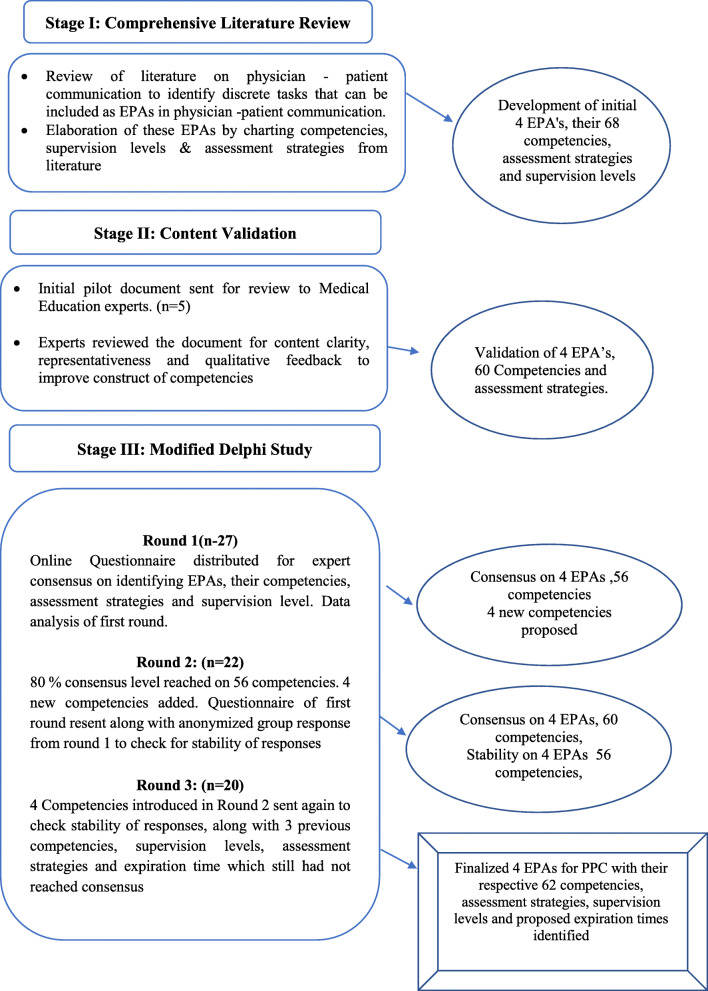
Stages of the study

This study was conducted in three stages (Fig. 1). The first stage was an extensive review of literature to identify the most specific physician-patient communication-related tasks that could be described as entrustable professional activities. The underlying competencies and assessment strategies for each of these EPAs were also identified. The second and third stages were both conducted as online surveys. The second stage was content validation by a small sample of medical Education experts (*n* = 5) for clarity and representativeness. The third stage was three iterative rounds of modified Delphi which were conducted with predetermined criteria of consensus described for every round. Items reaching consensus in the first round were sent in the second round to ensure stability of responses. In the last round, the few remaining competencies and assessment strategies were redistributed to experts for final consensus and stability of responses. This process is depicted in Fig. 1. The duration of the study was 9 months, including its conception, data collection, and reporting. The first stage of the study was completed in 4 months. The second stage involving content validation of the proposed EPAs was conducted in 2 months. The third and final stage of the study involving three rounds of Delphi was concluded in the next 3 months. Ethical approval for the study was obtained from the Ethical Review Committee of Riphah International University (Reference # Riphah/IIMC/IRC/20/134).

### Stage I: literature review

We conducted an initial review of the literature with the explicit purpose of identifying the communication-related tasks that could be considered EPAs for PPC. Various curricula implemented at different levels of undergraduate and postgraduate medical education were studied. National curricular guidelines in Pakistan related to teaching and assessment of communication skills were also reviewed. These guidelines recommend the integration of behavioral sciences into the undergraduate medical and dental curriculum [28]. Traditionally, communication skills are taught as part of the behavioral science curriculum in Pakistani medical universities [29, 30]. Thus, we also reviewed nationally recommended textbooks on behavioral sciences [31, 32]. Internationally, we reviewed published literature on communication skills curricula [15, 33–37] to identify our proposed EPAs. The literature review was further conducted on four databases and search engines, including ERIC; PubMed; PsychINFO & Google scholar to elaborate on the available evidence on the curricula, teaching methodologies and assessment strategies of each of these tasks in an attempt to quantify them as Entrustable professional activities. A snowballing approach was used as an addition to identify pertinent articles. We also reviewed EPA development literature [23, 25, 38] to ensure that the formats we developed for each EPA developed were according to the recommended guidelines [39].

Literature on the process and conduct of content validity was appraised [40, 41]. Lastly, we also reviewed literature on the modified Delphi process [42–44]. The literature review helped to develop a questionnaire with initial 4 EPA’s, 68 competencies, assessment strategies and supervision levels. No specific number of competencies for each EPA was predefined, and initially multiple competencies with almost similar meaning were selected to allow for subsequent revision or deletion. Published guidelines [45, 46] were consulted to ensure that grammatical and other errors were not present in the construct of the competencies.

### Stage II: content validation

The second stage was a content validity study meant to establish the clarity and to improve phrasing and understanding of the competencies developed for each EPA. Content validity measures how well items correspond or reflect a specific domain and are measured using quantitative techniques [47]. The competencies identified from literature were sent to 8 medical education experts who either had an extensive experience of developing curricula or were clinicians with an additional formal degree in medical education. All experts had Masters/Doctoral Degree in Medical Education, were serving in undergraduate/postgraduate/ CPD educational institutions and had a minimum experience of 5 years in curriculum development. Out of 8 experts initially contacted by email, 5 agreed to participate. Four of these experts were from Pakistan, while 1 international expert also participated (Table 1, pg22). The email included an introductory statement about the research study and its purpose. These experts reviewed each competency in these 4 EPAs identified from literature for Content Clarity & Representativeness (for internal content validation). Comment boxes were also provided for qualitative feedback. For Content validation, the items were graded for clarity to calculate content clarity average (CCA) and representativeness for I-CVI The clarity of an item is evaluated on the basis of how clearly an item is worded to consider the entire measure and specify the addition or deletion of any item [41]. The experts were asked to rate the competencies for both aspects by marking them on a 4-point Likert scale {(Clarity; 1 = item is not clear, 2 = item needs major revisions to be clear,3 = item needs minor revisions to be clear,4 = item is clear)(Representativeness; 1 = item is not representative 2 = item needs major revisions to be representative 3 = item needs minor revisions to be representative 4 = item is representative)}Space was also provided for open ended comments for qualitative feedback of each construct. The content validity survey was open for 4 weeks. This additional step helped to refine the framework of the EPAs. Content Clarity Averages (CCA) and Internal Content Validity (ICVI) were calculated separately for each of the competencies included in the initial questionnaire [41]. Changes in phrasing of the competencies were also made according to suggestions of the experts.

**Table 1:** Characteristics of Study participants

	Gender	Experience	Location
**Content Validation**	Males 40% Females 60% (*n =* 5)	5–10 yrs 20% 10–15 yrs 40% > 15 yrs 40%	Asia 80% Europe 20%
**Modified Delphi**	Males 55% Females 45% (*n* = 27)	5–10 yrs 26% 10-15 yrs 33.3% > 15 yrs 41%	Asia 74% Europe 3.6% Africa 7.8% North America 14.8%

### Stage III: the Delphi rounds

A questionnaire was then prepared incorporating all components of EPAs i.e., competencies, assessment strategies and supervision levels and designed on “Google Forms” for distributing, collecting and analysing the data. Literature on best practices in Delphi studies [42] suggests that consensus levels should be predefined, and descriptive statistics (mean/median and standard deviation/interquartile range) can be used to calculate consensus amongst an expert panel [48]. In this study, we used, percentage agreement, median and interquartile range using the following predefined criteria for consensus

- For Round 1, 2, and 3: ≥ 80% participants’ agreement in the top 2 measures (“extremely” or “very important”), median ≥ 4 and interquartile range ≤ 1 on a 5-point Likert scale
- The study would conclude on the achievement of consensus. Stability was calculated using McNemar change test by comparing responses of successive rounds [48, 49]. A significant *p*-value of < 0.05. suggesting instability with change of responses between rounds while a *p-*value > 0.05 would show consistent results with response stability.

### Study participants

Various experts around the world who fit the inclusion criteria were contacted via two social media platforms, LinkedIn and WhatsApp. Experts with demonstrated experience of developing and implementing communication curricula in the health professions were also sent personal message requests on LinkedIn requesting for their participation in the study. All participants were at least Assistant Professors or equivalent with a minimum teaching experience of 5 years in respective specialties. All either had a formal medical education degree or at least had done a certification course in medical education, and were actively involved in teaching and assessment of undergraduate medical students or postgraduate medical trainees. Twenty-seven experts showed their agreement to participate in the Delphi study. Participant specialties included general surgery, gynecology, pediatrics, medical education, dermatology, family medicine, psychiatry, anesthesiology, radiology, pathology, forensic medicine, rehab medicine and dental specialties including prosthodontics and maxillofacial surgery. Geographically 74% of participants were from Asia (Pakistan, Malaysia, Kingdom of Saudi Arabia) 14.8% from North America (U.S.A and Canada)7.4% from Africa (Egypt) and 3.4% from Europe (Switzerland) (Table 1).

### Delphi round I

The participants were asked to rate and comment regarding the importance of identified competencies for Physician -Patient Communication. Five-point Likert items were used for each EPA and their competencies, and participants were asked to rate each statement for the level of importance (“not at all important,” “slightly important,” “moderately important,” “very important,” “extremely important”). They were also required to choose relevant assessment strategies for each EPA and to identify desired supervision level by the end of postgraduate training. Participants were asked to provide any additional EPAs, competencies, assessment strategies or suggestions/feedback via comment boxes with every question. Participants were informed about the approximate time to complete the survey (20 to 30 min) and anonymity was ensured to minimize bias.

Statistical analysis of quantitative data was done using IBM SPSS Statistics 22 (IBM Corporation, USA). Percentage responses, median and interquartile ranges were calculated. Percentage ranking of assessment strategies and preferred patient satisfaction tool were also calculated and those with ≤20% consensus were excluded. Items reaching consensus were resent in round 2 to check for response stability. For supervision level, ≥80% agreement on a specific level was set as a criterion for inclusion. Qualitative data (input from the provided comment boxes) were also analyzed. No new EPAs were identified, however, suggestions regarding inclusion and further elaboration of few competencies were addressed in the second round by rephrasing for more clarity.

### Delphi round II

For Round II, individual emails were sent to each of the 27 experts who had participated in Round 1 to ensure their anonymity. The emails contained response sheets for each Delphi expert. Each sheet contained the entire Round II questionnaire, along with concerned experts’ answers for Round I. Anonymized group response for Round I was also mentioned in front of the individual response. The next column contained space for the expert’s response for Round II, and reason for change in response, in case of change of opinion.

The questionnaire comprised 64 competencies, grouped under 4 EPAs along with the supervision levels, assessment strategies and proposed expiration time for each of the EPAs. Round 2-survey remained active for 3 weeks. Data was collected as the participants replied to the emails. The aim for this round was to establish the stability of the responses, as consensus had been reached on almost all competencies of the EPAs.

Descriptive statistical analysis of quantitative data was done. Median and interquartile ranges were calculated along with percentage responses. To check the stability of the expert panel responses; inferential statistics were also performed. As the number of participants was less than 30, the McNemar Change test was used to see the stability of responses [49, 50] This test using chi-square statistics computes *p*-value, indicating a change of responses from one round to another when the responses are dichotomous. The null hypothesis was that responses have not significantly changed from one round to the other.

### Delphi round III

The Round three questionnaire contained certain competencies, supervision levels, assessment strategies and proposed expiration time for which either consensus had not been achieved in Round II or response stability had not been shown. All participants who had completed both the first and second rounds (*n* = 22) were again emailed individually. Items for which both consensus criteria had been met and response stability had been seen were excluded from the Round III-questionnaire. The aim was to check response stability and consensus for all remaining items. Round III remained active for 3 weeks. Participants were requested to mark the remaining competencies on the same Likert scale as in the previous two rounds. Previous individual and group responses were provided to them, in the same format as Round II. Again, an 80% agreement rate was used to accept or reject items from the final list. Participants were also asked to re mark the relevant supervision levels for each EPA. Consensus had yet to be developed on the top assessment strategies and expiration times for the EPAs. Percentage responses were calculated and pre-defined agreement rate was used to include items in the final list of EPAs and their competencies. The remaining competencies, supervision levels, assessment strategies and proposed expiration time were all evaluated for stability of panel responses by applying the McNemar change test again and applying the null hypothesis criteria used in Round II.

## Results

### Stage I: literature review

Our literature search enabled us to identify explicit physician patient communication related tasks that could be defined as acts of entrustment. We tried to ensure that each of these tasks were discrete and not mutually exclusive [38, 51] and were focused on specific interactions between the physician and patient according to the contextual environment. This thorough search of the literature helped identify the following tasks that could be developed into Entrustable professional activities.

1. Providing information to the patient about his diagnosis [52–56]
2. Breaking bad news to a patient/his family [57–64]
3. Counseling the patient regarding his disease [65–67].
4. Resolving conflicts with patients or their families [68–72]

We further performed a methodological search for literature available on the available curricula, teaching and assessment strategies for each of these EPAs. Our search enabled us to develop an initial EPA document, with 4 EPAs 68 competencies, proposed assessment strategies and supervision levels for these EPAs on Physician- patient communication.

### Stage II: content validation

Five experts agreed to participate in content validation. Clarity and representativeness were calculated using 5 raters on a 4-point Likert scale. Overall, Average clarity scores for CCA less than 3.5 were considered for revision and those with CCA less than 3 were discarded. Individual Content Validity Index (I-CVI) for the competencies in the 4 EPAs were calculated on the basis of their representativeness. Competencies with I-CVI less than 0.70 were eliminated. Those with I-CVI between, between 0.70 and 0.79, were revised, and I-CVI values > 0.79, were retained as such without any changes. However, qualitative expert feedback was used for final decision making, regarding placement and construct of the competencies. Content validation resulted in refinement of the 4 EPAs, their competencies along with their established supervision and entrustment scales for postgraduate medical education.

Content validation of the EPAs for PPC modified the construct of 2 EPAs, and 30 competencies. Final EPA document comprised 4 EPAs and 60 competencies were finalized to be sent to the experts for consensus building in the 1st round of Delphi.

### Stage III: the Delphi rounds

#### Delphi round I

Twenty-seven experts (77%) out of 35 initially contacted experts participated in Round I. Analysis of Round 1 results showed that out of the 60 competencies included in 4 EPAs, only 4 did not meet consensus criteria. The remaining 56 competencies met the predetermined criteria for consensus. Consensus was also not met for the supervision levels of all 4 EPAs, assessment strategies, and proposed expiration time. Qualitative feedback was reviewed by all authors and changes were made in the construct of EPA-I and some competencies to ensure gender-neutrality and diversity. Qualitative Feedback resulted in increasing the total number of competencies to 64. It was decided to resend the entire Round I questionnaire to the experts who had consented to participate in Round I, along with few newly added competencies for online communication. Expert responses would be checked for stability (for all competencies, those, which had met consensus and those which had not) while consensus would be sought for the supervision levels, assessment strategies and expiration time of the EPAs.

#### Delphi round II

The Round II-questionnaire was emailed to the 27 experts who had participated in Round I. It contained the previous 60 competencies, 4 new ones and several statements (EPA-1 phrasing & competencies) that had been modified or elaborated because of qualitative feedback. Round 2 remained active for 3 weeks; reminder emails were sent at the end of each week to experts who had not submitted their responses. This helped increase the response rate. After 3 weeks, 22(82%) responses had been collected and Round II was closed. Consensus criteria for inclusion in Round II were the same as Round I. Analysis of Round II results revealed that consensus was again achieved on 56 competencies for the 4 EPAs. The 4 new competencies which had been introduced in Round II and 3 of the previously undecided competencies had also achieved consensus but would be sent again in Round III to check stability of responses. The supervision levels, remaining assessment strategies and validated checklist/scale had still not reached a consensus. Also, the proposed expiration time of the EPAs was not agreed upon. To confirm the stability of responses, the McNemar change test was applied. It showed the stability of maximum responses with *p* value > 0.05 for all initially selected 56 competencies.

#### Delphi round III

In Round III, the competencies which had either been introduced in Round II, and those that had not achieved 80% consensus were resent to 22 experts for final review along with the supervision levels, assessment strategies and proposed expiration time for the EPAs. Previous individual and group responses were also sent to the experts. Percentage agreement was sought for all items in the questionnaire for Round 3. 20 experts (91%) participated in Round III. Results of round 3 revealed that 80% consensus was again achieved on all competencies except one, Mean, median and IQR scores for this competency also did not reach the preset criterion and thus it was removed. At the end of round III, final 4 EPAs with collectively 62 competencies (Additional file 1) and assessment strategies (Table 2) all aiming at supervision “level 5” (Table 3) were identified after expert consensus. Consensus was also reached on the proposed expiration times (Table 4).

**Table 2:** Proposed supervision levels of EPAs for physician patient communication at the end of Round III

	Level 1: Be present & observe	Level 2: Act with direct, pro-active supervision, i.e. with a supervisor physically present in the room	Level 3*:* Act under indirect supervision, supervisor distantly available Findings reviewed	Level 4: Act under Distant supervision not directly available ***(unsupervised)***	Level 5: Provide supervision to junior trainees
**PG-yr1**	90%* 95%** 95%*** 95%****	10%* 5%** 5%***			5%****
**PG-yr 2**	5%* 5%*** 10%****	90%* 95%** 95%*** 90%****	5%*		5%**
**PG-yr 3**	5%* 5%** 10%****	5%* 10%** 5%***	75%* 80%** 85%*** 85%****	5%*	5%* 10%*** 5%****
**PG-yr 4**		10%** 10%****	5%* 5%** 5%***	80%* 80%** 85%*** 80%****	15%* 5%** 10%*** 5%****
**PG-yr 5**		5%****	10%** 5%*** 5%***	5%* 5%** 5%*** 5%****	95%* 85%** 90%*** 85%****

*EPA 1: Providing information to the patient or their family about the patient’s diagnosis and prognosis; **EPA 2: Breaking bad news (BBN) to a patient and/ or their family; ***EPA 3: Counsel the patient regarding their disease; **** EPA 4: Resolving conflicts with patients or their families (Highest levels of agreement are highlighted in bold)

**Table 3:** Percentage Ranking of Proposed Assessment strategies at the end of Round III

Assessment Strategy	EPA1	EPA2	EPA3	EPA4
OSPE station utilizing standardized checklists & tools	5%	10%	5%	5%
Faculty Observation & feedback with standardized patients (SP)	5%		5%	5%
Faculty Observation & feedback with real patients	15%	15%	10%	15%
Combination of all strategies	**75%**	**75%**	**80%**	**75%**

**Table 4:** Proposed Expiration Time of EPAs at the end of Round III

Proposed Expiration time	2 yrs	3 yrs	4 yrs	No expiration
Providing information to the patient about their diagnosis/ prognosis		5%	15%	**80%**
Breaking bad news to a patient	10%		**80%**	10%
Counselling a patient regarding their disease	5%		**90%**	5%
Resolving conflicts with patients or their families			**90%**	10%

This study had high content validity ensured not only through the content validation process, but also by involvement of a reasonably large number of experts who had knowledge and experience of physician-patient communication, a high response rate of experts, the use of successive rounds and high criteria of response agreement.

## Discussion

To our knowledge, this is the first study of its kind in which the EPA approach is being used to outline competencies and assessment tools for a patient-physician communication course, thus creating an entrustment framework for resident supervision. The EPAs for Physician-patient communication are a step toward an integrative, all-inclusive competency-based assessment framework for patient-centered care. They are meant to improve the quality of physician patient interaction by standardizing communication as a decision of entrustment. Each EPA developed by expert consensus is a separate, discrete, observable and measurable task. Thus, it fits the description of an EPA as defined by Ten Cate [39]. Entrustment for these EPAs require proficiency in numerous communication competencies which can be assessed by several workplace- based assessment tools. Consensus for suitable assessment tools was also achieved during this study.

Recent literature on EPA’s development [73] provides detailed guidelines on the kind of tasks that can be selected for developing an EPA. The authors advocate that an EPA should not be too broad and should allow for feasible assessment and focused entrustment. Considering all tasks in physician- patient communication as one whole would therefore not be feasible as it would be difficult to assess the learner for all the tasks involved in patient communication. For eg: A year 1 PG trainee could be entrusted with providing information to a patient about his diagnosis, but he may not be able to demonstrate the competencies needed for breaking bad news or resolving conflicts with patients too. Also, various other EPAs identified in the literature may also have overlapping knowledge, skills and attitudes [74]. This is because of the particular knowledge and skill base developed by a student in the early years of medical training will enable them to develop cognitive schemas [75], which will aid in clinical decision making for various tasks during clinical practice and not necessarily a single task only.

A recently published article on EPAs [73] purports the use of a construct validity lens for EPA development, to ensure that EPAs accurately reflect the work of a profession or a specialty. This article has been published after the conduct of this study. However, it is interesting to note that the guidelines provided by the authors for ensuring the validity of the newly developed EPAs are similar to the steps followed in this study. The authors in this article state that expert selection is a crucial process in the in determining the content of the EPAs. Three major themes come into consideration here, first; is the inclusion of experts from different domains [73] In this study, the content validation process has helped ensure that the EPA document was also viewed by educational experts with specific experience in curriculum and assessment before the involvement of clinical experts during the Delphi. This helped get validation from medical education experts on the placement of these competencies within each EPA [47]. Second; is that by using a purposeful sampling technique and trying to ensure that experts from major medical and dental specialties were included in this study, representativeness was established to some extent and bias was minimized. Third; by providing clear instructions to the experts about the development methods and their role during content validation and Delphi, confusion and misinterpretation of the content was avoided.

Another recent article by Tekian et al. [76] discusses certain strategic points for implementing EPA’s based curricula, and notes that the entrustment of an integrated task or activity cannot be done until there is an understanding of the essential components, which make up that activity. Keeping in mind this concept, each EPA for physician patient communication has been developed in such a way, that the entire task or activity has been broken down into a series of competency domains, which describe the desired abilities of graduates. This creates a mental model that will facilitate both learners and assessors during the implementation of the EPA ensuring that the benefits of CBME are also fully exploited. Another reason is the prior intention for developing these EPAs to be adaptable to various postgraduate specialties. Thus, wherever the EPAs for PPC are implemented, curriculum developers will have in hand an evidence-based format to aid them.

The EPAs for physician- patient communication provide observable, measurable activities for patient-centered communication that can be linked to competency frameworks around the world. They can provide a useful assessment framework for effective training in patient communication. These EPAs identify the most common communication related entrustment decisions that require fulfillment by graduate trainees or residents working in every clinical specialty. They can be integrated into any post graduate curriculum and contain the desired competencies related to knowledge, skills and attitudes as well as suggestions for assessment strategies. Literature on physician patient communication identifies the educational strategies, and instructional tools needed for each EPA [36, 77, 78]. Similarly, Expert consensus shows that entrustment would require a combination of assessment tools to assess these EPAs. Faculty feedback has also been agreed upon for assessment and must ideally be conducted utilizing standardized communication specific checklists.

These EPAs may also serve as a self -assessment tool for postgraduate training programs across the globe to improve their patient communication curricula. By defining specific physician-patient communication tasks in the language of EPAs, we can guide both the students and teachers in their respective roles and develop an exclusive physician-patient communication course that can be used not only in postgraduate education but also for continuing professional development in patient communication.

### Limitations of the study

An important limitation of this study is that for high stake EPAs, which lead to unsupervised practice, literature suggests that expert panels for EPA development should ideally also include, patients, learners and allied health professionals [73]. We were, however, unable to solicit patients who would be able to understand the complex nature of these EPAs and participate in our study to make a useful contribution. Developing a detailed curriculum for these EPAs to link the competencies to milestones was also out of the scope of this study. The limitations of each EPA along with desired performance standards or the milestones also need to be determined.

Our results also showed that experts agreed that a combination of assessment methods, should be used for these EPAs. It must be reiterated that communication is a multifactorial competency and thus assessment of these EPAs should also utilize multiple assessment strategies, ideally in combination with other clinical EPAs rather than in a standalone manner. Several assessment tools were also ruled out during consensus, which we believe could potentially be beneficial during application. Thus, further research piloting and evaluation should help to improve the implementation of these EPAs before formal adoption into a program of study.

Another limitation of this study is that the validity evidence focused only on content validity. Further research, piloting and evaluating these EPAs is suggested for obtaining additional validity evidence. Some studies have also used internally validated tools to measure the quality of the developed EPAs [79] Others have also used rubrics [80] for alignment with purpose. Due to lack of time, we were unable to use either of these formats to evaluate these EPAs.

### Conclusion

This study has resulted in the development of 4 EPAs with collectively 62 competencies, all aiming at supervision “Level 5” by the end of postgraduate resident training along with various assessment strategies. The EPA approach for physician patient communication makes it a valuable approach where the major goal of optimal patient care remains in clear sight.

The need for improvements in teaching and assessment of physician- patient communication has been highlighted in various national and international studies. This is the first study of its kind, which has not only first gotten validation from expert medical educationists for the content of each EPA, but after that has achieved desired consensus from various national and international experts.

The Delphi experts in this study were of predominantly Asian origin, however, the content of these EPAs is such that they are generalizable to diverse cultural backgrounds. Recreating this study in another cultural context, would however be interesting to note and could provide insight into how entrustment of communication related tasks can vary across societies. The authors thus recommend that health communication experts from different cultural backgrounds should be solicited to review these EPAs, enabling further refinement before implementation.

These EPAs have been developed in such a way that they can be applied side by side with any postgraduate program wherever trainees are expected to communicate with patients daily. By defining competencies and learning objectives in the language of EPAs, this study can provide a roadmap and a source of explicit guidance for both students and faculty in identifying the goals and expectations for physician- patient communication.

It is hoped that this study will provide a basis for universal acceptance of communication as an act of entrustment and help to provide guidelines for teaching and assessment of Physician patient communication.

## Supplementary Information

**Additional file 1. **Percentage agreement and *p*-values for final competencies included in each EPA at the end of Round III. 1. The data set shows mean percentage agreement of experts on competencies included in each EPA at the end of round III of the Delphi process. 2. *p-*values for each competency, calculated by applying the McNemar test to check for response stability in two successive rounds.

## Data Availability

The datasets used and/or analysed during the current study are available from the corresponding author on reasonable request.

## Abbreviations

EPA: Entrustable Professional Activities
CBME: Competency Based Medical Education
PPC: Physician -Patient communication
CPD: Continuous Professional Development
CCA: Content Clarity Average
I-CVI: Individual Content Validity Index
