# Supplementary material for: Entrustment in physician-patient communication: a modified Delphi study using the EPA approach
Source: BMC Med Educ. 2021 Sep 20;21:497. doi: 10.1186/s12909-021-02931-1 (PMC8454034; doi:10.1186/s12909-021-02931-1)
Supplement: Supplementary file 1 — Additional file 1. Percentage agreement and p-values for final competencies included in each EPA at the end of Round III. 1. The data set shows mean percentage agreement of experts on competencies included in each EPA at the end of round III of the Delphi process. 2. p-values for each competency, calculated by applying the McNemar test to check for response stability in two successive rounds. [file 12909_2021_2931_MOESM1_ESM.pdf]

Table : Percentage agreement and p-values for final competencies included in each EPA at the end of Round III

	**EPA1: Providing information to the patient or their family about the patient's diagnosis and prognosis**	**% agreement (mean value)**	**p-value**
1	Know the complete medical picture of the patient, necessary for their diagnosis including history, current condition, required investigations, treatment options, and prognosis	100	*
2	Choose the most appropriate PPC model for conveying verbal information to the patient	91	*
3	Choose the most appropriate PPC model for conveying written information to the patient	96	0.065
4	Justify an evidence-based approach for choosing the most appropriate PPC model according to the presenting situation	81	1.00
5	Utilize nonverbal cues (body posture & facial expressions) to make the patient comfortable before delivering relevant information.	91	*
6	Communicate the relevant diagnosis and/ or prognosis clearly to the patient in physical proximity	100	1.00
7	Communicate the relevant diagnosis and/ or prognosis clearly in online consultation for telemedicine	100	*
8	Explain complex information in a manner comprehensible to the patient (using diagrams, pictures etc)	100	1.00
9	Assess patients’ understanding of treatment options for the concerned diagnosis and /or prognosis	100	1.00
10	Ensure that the patient is able to express his/ her concerns and preferences and make personal choices	100	1.00

11	Respond to patients' queries or concerns about diagnosis and prognosis	100	1.00
12	Show understanding & empathy towards the patient and their condition	100	1.00
13	Recognize the patient's emotional state by noting his/her verbal and nonverbal gestures	91	1.00
14	Allow sufficient time for patient to display emotions if required	87	1.00
15	Respond appropriately to patients' ideas & feelings about his/her diagnosis and prognosis	100	1.00
16	Exhibit the capacity to clarify any miscommunication relating to the prognosis/ diagnosis	100	1.00
	**EPA -2: Breaking bad news (BBN) to a patient and/ or their family**		
1	Appraise the complete case history of the patient including his/ her current condition and prognosis.	96.2	0.5
2	Differentiate various communication models for breaking bad news (BBN) according to the severity of the situation.	85.1	1.00
3	Rationalize an evidence-based approach for choosing the most appropriate model of BBN	85.1	1.00
4	Demonstrate effective listening by using non-verbal cues.	81.4	1.00
5	Listen in a way that patient feels heard and understood.	92.5	1.00
6	Assess patient's or family's understanding of his/her condition	96.2	1.00
7	Clarify using words that are easy for the patient /family to understand	96.2	1.00
8	Demonstrate the ability to clarify any miscommunication with patient/ family	99.9	1.00

9	Develop a supportive atmosphere for the patient/ family by encouraging patient participation and involvement in decision-making	96.2	1.00
10	Include patient/ family in choices and decisions to the extent he or she desires.	85.1	1.00
11	Display empathy towards the patient / family and their situation	96.2	1.00
12	Respect the patient's cultural and ethnic beliefs, practices and language	96.2	1.00
13	Handle disagreements with patients/families courteously	99.9	1.00
14	Respect patients'/ families' values and opinions while framing decisions for future action	92.5	1.00
	**EPA-3 Counsel the patient regarding their disease**		
1	Understand the complete condition of the patient necessary for counselling him/her, including history of current illness, required investigations, treatment options, and prognosis	96.2	1.00
2	Select the most appropriate model for counselling the patient about their disease according to his/ her present situation	88.8	1.00
3	Rationalize an evidence-based approach for choosing the most appropriate problem-solving strategy for effective interventional counselling	90	0.125
4	Develop a clearer understanding of patient's health related issue based on principles of best evidence medicine.	88.8	1.00
5	Identify the various choices and options available to the patient allowing them to express concerns and preferences.	92.5	1.00

6	Propose acceptable & explicit goals that the patient is willing to achieve as a result of the counselling session	88.8	1.00
7	Use Vocal communication skills (including volume, articulation, pitch, emphasis, speech rate &use of pauses and silences) to reduce patient anxiety in both physical proximity and online consultation	92.5	1.00
8	Utilize nonverbal communication skills (facial expression, eye contact, Posture, etc) for making the patient comfortable in both physical proximity and online consultation	81.4	1.00
9	Communicate with the patient professionally in an online environment	100	*
10	Establish a relationship of mutual trust and care in which patients and their families feel secure and able to express themselves	88.8	1.00
11	Attend to the patient with purposeful consideration, allowing him/her to seek clarification of terms, issues and misgivings	95	1.00
12	Reflect and paraphrase the patient's story	81	1.00
13	Recognize the patients emotional state keeping note of both verbal and nonverbal cues	92.5	1.00
14	Use words and phrases that are easy for the patient to understand	88.8	1.00
15	Demonstrate respect for patient privacy and autonomy	96.2	1.00
16	Demonstrate sensitivity, honesty, and compassion in difficult conversations	96.2	1.00
17	Maintain appropriate boundaries and relationships with patient to avoid fostering dependency	88.8	0.5
18	Allow sufficient time for patient to display emotions if required	88.8	1.00

19	Ensure that the counselling session results in a shared decision-making process which is in the patients' best interests	85.1	1.00
	**EPA 4: Resolving conflicts with patients or their families**		
1	Explain various models of conflict resolution that may be applied to understand and resolve conflict situations during Physician-patient communication	95	0.50
2	Select the most appropriate conflict resolution model according to the presenting circumstances	92.5	0.625
3	Justify an evidence-based approach for choosing the most appropriate model	95	1.00
4	Adapt a variety of techniques, including nontechnical language, appropriate pacing, and small pieces of information to ensure that source of conflict with patients /family is clarified to all	92.5	0.625
5	Ensure that the patient/ family is able to express concerns and preferences and make personal choices	92.5	0.625
6	Respond to patients' queries or concerns that have risen due to the conflict situation	85.1	1.00
7	Involve mediators (supervisors, administrators, ethicist) for resolution of conflict when and if required)	92.5	1.00
8	Demonstrate confident and understanding attitude that puts patients & their families at ease	88.8	1.00
9	Ascertain the emotional state of the patients/families keeping note of both verbal and nonverbal gestures	96.2	1.00
10	Display respect for patient privacy and autonomy	92.5	1.00

11	Approach difficult patients with tolerance, patience, utilizing relaxation and calming techniques	96.2	1.00
12	Display effective conflict resolution skills aligned with enlarged interests of self and patient, resulting in solution acceptable to all	92.5	1.00
13	Recognize one's own limitations as party to the conflict situation and request involvement of mediators for conflict resolution	92.5	1.00

*Could not be computed because 2 data sets are exactly the same
